# Urban Intersection Classification: A Comparative Analysis

**DOI:** 10.3390/s21186269

**Published:** 2021-09-18

**Authors:** Augusto Luis Ballardini, Álvaro Hernández Saz, Sandra Carrasco Limeros, Javier Lorenzo, Ignacio Parra Alonso, Noelia Hernández Parra, Iván García Daza, Miguel Ángel Sotelo

**Affiliations:** Computer Engineering Department, Universidad de Alcalá, 28805 Alcalá de Henares, Spain; alvaro.hernandezsaz@uah.es (Á.H.S.); sandra.carrascol@uah.es (S.C.L.); javier.lorenzod@uah.es (J.L.); ignacio.parra@uah.es (I.P.A.); noelia.hernandez@uah.es (N.H.P.); ivan.garciad@uah.es (I.G.D.); miguel.sotelo@uah.es (M.Á.S.)

**Keywords:** intersection classification, scene understanding, self driving, intelligent transportation systems, CNN, GAN, RNN

## Abstract

Understanding the scene in front of a vehicle is crucial for self-driving vehicles and Advanced Driver Assistance Systems, and in urban scenarios, intersection areas are one of the most critical, concentrating between 20% to 25% of road fatalities. This research presents a thorough investigation on the detection and classification of urban intersections as seen from onboard front-facing cameras. Different methodologies aimed at classifying intersection geometries have been assessed to provide a comprehensive evaluation of state-of-the-art techniques based on Deep Neural Network (DNN) approaches, including single-frame approaches and temporal integration schemes. A detailed analysis of most popular datasets previously used for the application together with a comparison with ad hoc recorded sequences revealed that the performances strongly depend on the field of view of the camera rather than other characteristics or temporal-integrating techniques. Due to the scarcity of training data, a new dataset is created by performing data augmentation from real-world data through a Generative Adversarial Network (GAN) to increase generalizability as well as to test the influence of data quality. Despite being in the relatively early stages, mainly due to the lack of intersection datasets oriented to the problem, an extensive experimental activity has been performed to analyze the individual performance of each proposed systems.

## 1. Introduction

**Figure 1:**
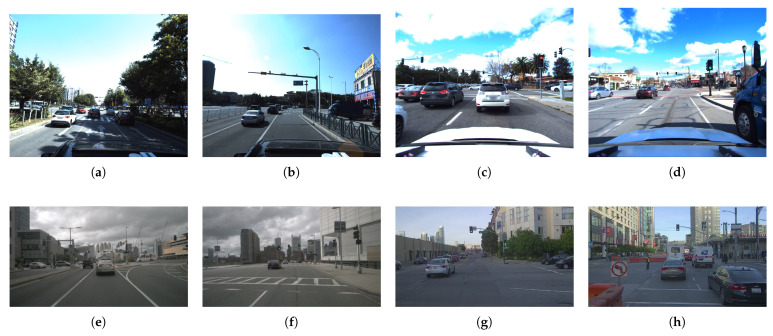
Typical intersections in the well-known ApolloScape (**a**,**b**), Lyft-5 (**c**,**d**), NuScenes (**e**,**f**), and Pandaset (**g**,**h**) datasets. The intersection geometry configuration on these Manhattan-like environments is simple and repetitive, and the dominant difference consists only on the traffic conditions. Moreover, it is of extreme difficulty to generalize these kinds of settings to mid-sized cities.

**Figure 2:**
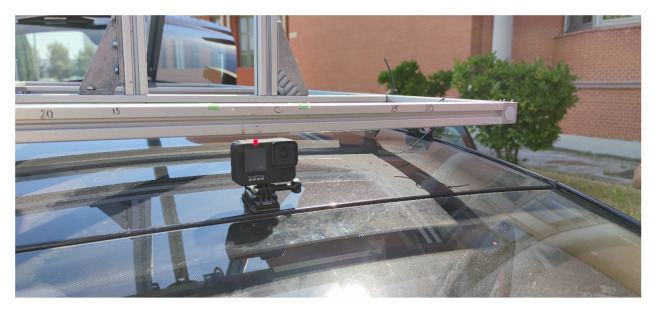
Our vehicle with the attached consumer action camera.

Estimating the scene in front of a vehicle is crucial for safe autonomous vehicle maneuvers, and it is also key to Advanced Driver Assistance Systems (ADAS). Even though performance and availability of scene understanding systems increased over the past years, technology seems to be far from the requirements of SAE (https://www.sae.org/standards/content/j3016_202104, accessed date 16 August 2021) full automation level, particularly regarding urban areas and contexts without a strict Manhattan-style city plan, i.e., large building blocks surrounded by simple straight avenues. The transition towards full automation requires reliability under all circumstances, including ordinary middle and small cities where narrow and strangled roads are the most common. In 2019, approximately 22,700 people died throughout Europe due to traffic accidents according to the European Commission’s latest report [1], and the last Annual Accident Report of 2018 [2] show us that the 19.4% of EU road fatalities comes from at-grade intersection areas. Similar results come from the reports of the United States National Highway Traffic Safety Administration (NHTSA), which show us that in the 2015–2019 period, at-grade intersections areas concentrate more than 40% of motor vehicle and 25% of fatal crashes [3]. Indeed, intersection areas are one of the most critical for both drivers and pedestrians. It is where vehicles’ paths might cross at some point and where pedestrians are most likely to cross the roadway. Consequently, within the navigation system of an autonomous vehicle, it can be considered of paramount concern to have a reliable system able to identify this specific traffic scene. For example, the vehicle control system might notice the number of road entries to the upcoming crossing and therefore estimate from which trajectories can come either other vehicle or pedestrian on a collision course. Besides the safety field, knowing the topology of the upcoming junction can allow the vehicle to perform safe maneuvers without the need to have a complete and detailed map of the area, as a human being would do when asking for directions in a city he does not know. In addition, we also believe that we can exploit the detection and classification of intersections for multiple purposes, such as an input to high-level classifiers of other driver’s maneuvers. This might ease the prediction of position and intentions of Vulnerable Road Users (VRU) [4], or plan how to drive centered and safely without road markings. Toward this goal, existing intersection detectors were often paired with localization algorithms coupled with an external Global Navigation Satellite System (GNSS) and map data coming from providers like TomTom or HERE, which started to provide maps commonly referred to as High-Definition maps (HD maps). Even though the benefit of a priori knowledge on the understanding of the surrounding environment is undisputed, as it allows the system to narrow the scene reconstruction uncertainty, we also argue that relying only on updated maps might jeopardize the safety of autonomous driving systems themselves. Given the impact of vehicle crashes on human lives and the plethora of driving scenarios needed to consider, it follows that autonomous driving cars should be able to overcome, if not all, at least most of the common driving events that arise in actual traffic conditions. This work presents an in-depth study of the intersection classification problem and, following previous literature contributions, we defined seven basic geometries to which we will refer throughout the remainder of our contribution. Toward this goal, we explored the capabilities of state-of-the-art neural network approaches for image classification, considering different approaches such as single-frame, video analysis, and 3D-networks; non-traditional learning techniques for the specific intersection analysis subject; as well as different data source, e.g., RGB cameras and Light Detection And Rangings (LiDARs) sensors. The availability of intersection data is crucial in the Deep Neural Network (DNN) context, and both industries and research entities have worked hard during the last years to create large datasets explicitly conceived for autonomous driving research purposes. However, most of these datasets are collected in extensive metropolitan areas, in which ample avenues are the majority. On the one hand, tackling the classification problem in these scenarios is usually of extreme difficulty due to mere visibility issues, see Figure 1, that typically arise in these conditions. Intersections areas on these specific scenarios are generally well defined and thoroughly regulated with horizontal lane markings and traffic lights, circumstances that high-level self-driving systems can exploit for their advantage. This intuition was confirmed after a comprehensive yet intensive survey on the most popular self-driving datasets publicly available, including ApolloScape [5], Argoverse [6], Lyft-5 [7], nuScenes [8], and Pandaset [9]. On the other hand, intending to provide similar aid to the same high-level systems but in less structured environments, in this paper we delve into the identification of intersections in less metropolitanized areas. Note that well-known KITTI [10] and KITTI-360 [11] datasets, even though collected in the mid-sized city of Karlsruhe, Germany, contain an extremely low number of intersections, in the order of a few hundreds, including all the *City* and *Residential* sequences of the Raw-Data section. Moreover, the focal length of the cameras used in this dataset makes the recognition task harder, as, over short distances, it is not easy to see the whole intersection (whereas over long distances intersecting streets are unnoticeable). Last, this dataset contains only images with similar lighting conditions. For this reason, we decided to record a new dataset in the cities of Alcalá de Henares and Meco, Madrid, Spain. This dataset contains more than 530 intersections in different light conditions, more than all of those contained in the overall KITTI and KITTI-360 sequences. Concerning the camera configuration, we opted for a consumer action camera mounted on different vehicles as visible in Figure 2 that provided excellent visibility of the surrounding area, good lighting handling, and easy operation. To further increase the number of intersections during the training phase, we also exploited the generative capabilities of state-of-the-art Generative Adversarial Networks (GANs) [12] architectures, introducing new photorealistic images of intersections. Extensive experimental evaluations show the pros and cons of each of the different investigated techniques. The remainder of this work is organized as follows. Section 2 provides an overview of the existing literature on intersection classification. Section 3 presents the datasets used in this work along with technical details that highlight why we proposed a new intersection dataset. Section 4 introduces the techniques proposed in this work, and Section 5 presents the experimental activity and the ablation study that allowed us to analyze the performances of the proposed techniques. Finally, in Section 6, we present our conclusions and the future works.

## 2. Related WorkWord

The relevance of road intersection detection can be noticed from the interest in the problem coming from different research communities and traffic regulation agencies [13,14]. From a technical perspective, we can first distinguish approaches that exploit images from stereo or monocular camera suites, algorithms that only rely on LiDAR sensors, or finally, a combination of the previous. One of the earliest works in this research field can be found in [15], where two standard computer vision methods are presented. The authors proposed a first method to extract road boundaries from the images, matching them to a predefined intersection model using heuristic methods, followed by a second system aimed at finding free space corridors through which it is safe to drive. Later, in 2013, Andreas Geiger published his Ph.D. thesis [16] in the field of urban scene understanding. In his thesis, he proposed two new approaches designed for detecting and classifying intersection areas that, in many cases, can be considered the starting point of the recent research on this field. The first approach consists of a new intersection modeling system with flexibility in the number of intersecting streets and crossing branches’ location, orientation, and width. The second proposal involves an efficient learning and inference algorithm for scene understanding, based on Markov Chain Monte Carlo Sampling and belief propagation. In different contexts but with a similar approach, the authors of [17,18] exploited RGB images from vehicle front-facing cameras and standard computer vision techniques to create temporally integrated occupancy grids that were in turn compared to predetermined shapes to assess the presence of upcoming intersections. The approach proposed in [19] is substantially different. Here, the input is solely based on point clouds obtained by LiDAR sensors. These raw data go through three independent processes that generate a binary classification of whether they belong to an intersection or not. The first process is aimed to extract the most relevant features from the point clouds. These features capture appropriate geometric and statistical properties that are consequently classified through different classifiers, such as Multilayer Perceptron (MLP), Support Vector Machine (SVM), and AdaBoost. Finally, as these methodologies produce a single frame classification, the third step involves Hidden Markov Model (HMM) and Conditional Random Field (CRF) approaches to model the observation time series. Despite working with different kinds of sensors, i.e., LiDAR instead of cameras, the work presented in [20] is closer to our research goals and uses Convolutional Neural Networks (CNNs) approaches coupled with transfer learning techniques to classify intersections. Starting from a Fully Convolutional Neural Network (FCNN) trained to predict the future path of the ego-vehicle, the authors proposed an architecture called Intersection Net that exploits the encoder’s features to perform the intersection classification in terms of six basic classes, including 3-way, 4-way, and roundabouts. So far, the presented research based on deep learning has focused on detecting or classifying intersections once they have been reached. However, it has been seen in [19] that temporal integration can be an asset to be considered. In [21], this research line is further investigated and a Long Short-Term Memory (LSTM) [22] architecture is attached to a CNN to solve the *“Have I reached the intersection?”* problem. The difference between LSTMs and other types of architectures is that they can perform temporal integration thanks to a “short-term memory” and a “long-term memory” that allows them to remember what they have previously processed to execute the current prediction better. The workflow of their final architecture is as follows. First, a sequence of ordered images is supplied to the CNN, which will in turn return a sequence of feature vectors. These vectors are packed and sent to a LSTM network, which integrates them temporarily to extract information to binary classify the sequence as “intersection” or “not intersection”. From a technical perspective, our work extends theirs in the following ways. First, our work is aimed not only towards a binary classification task, but, following previous state-of-the-art approaches, we identified a broader set of intersection classes, even larger than their final three-class expansion. We also deep investigated different recurrent network architectures as well as fusion layers on a much larger set of data, to systematically evaluate the performance given by each different component. Last, it is important to notice that the authors highlight the lack of an existing dataset specifically designed and publicly available for evaluating the performance of an intersection detection method. Together with the KITTI dataset, our proposal address this issue, as we believe that much more data and research is needed in this field. The work presented in [23] maintains a similar approach about temporal integration, but instead of detecting whether the intersection is reached, it tries to classify them into seven basic typologies. For this purpose, their system uses two data sources that the authors called Input-F and Input-T. Input-T is an RGB image taken just before reaching the intersection, and Input-F is a sequence of images taken while crossing the intersection. The authors used an architecture composed of three different modules, F-Net, T-Net, and I-Net to process these two data sources. In detail, T-Net is a VGG16 network in which the classifier is replaced by two fully connected layers trained to distinguish between the seven classes, freezing the other weights. F-Net instead is a combination of Inception-V3 and an LSTM architecture. The goal of Inception-V3 is to extract the optical flow from the image sequence so that the LSTM can classify them between straight crossings, right-facing crossings, and left-facing crossings. Finally, I-Net is the architecture in charge of integrating the previous two architectures’ results to return a final classification using Bayesian multimodal information fusion [24]. Conceptually, some of the approaches are also investigated in our work, i.e., the use of LSTMs or VGG-based systems. However, the remainder of our work shows how better performances can be achieved by using simpler system configurations that do not require multi-level architectures to perform the intersection classification.

### 2.1. LSTM

Long Short-Term Memory networks are a type of Recurrent Neural Network (RNN) developed by Hochreiter et al. [22] to avoid vanishing gradient problems that are intrinsic in the vanilla RNN. This architecture was initially conceived for Natural Language Understanding and time series prediction tasks. However, in the last decade, they have been introduced in the computer vision world in combination with convolutional architectures. In [25], the authors proposed this combination to deal with the problem of image captioning (i.e., generating sentences describing an input image). Following this work, new models emerged adopting this combination of LSTM and CNN networks to the spatio-temporal domain of video sequences. In [26], a model called Long-term Recurrent Convolutional Networks (LRCN) is presented. This model combines a CNN backbone for spatial feature extraction from raw video frames and an LSTM network for temporal feature extraction. By summarizing the features obtained with the recurrent network, a final action is inferred for the whole input sequence. While this model is initially applied to human action recognition, in [21] this combination of networks is applied to binary intersection detection (i.e., probability of an intersection in the input video). A similar model is used in [27], but combining optical flow information with the raw video sequence, improving state-of-the-art results in several action benchmarks. As LSTM networks are not suitable for high-dimensional data due to their fully-connected nature, in [28] ConvLSTM was proposed. In this model, LSTM input-to-state and state-to-state transitions are replaced by convolutional architectures, making it suitable for image data. This model has been applied to several tasks such as medical image segmentation, video frame interpolation, and super-resolution. However, the LSTM standard model continues to be the standard among recurrent models and video action recognition is currently dominated by CNN 3D models.

### 2.2. GAN

It is a widespread practice to augment image-based datasets in ways that are appropriate for a given dataset. In general, data augmentation helps to stop a model from overfitting to the data and allows users to make small datasets many times larger. However, a sufficiently powerful classifier often still overfit to the original examples, which is why GANs are particularly useful here. GANs are well known for their utility in generating fake data when real data is either too hard to come by or expensive to acquire. One of the benefits of using GAN data augmentation is that they are often better than handcrafted synthetic examples. Another pro of GAN data augmentation is that it can generate labeled examples using a conditional setup, which is really useful in case of imbalanced datasets. Finally, it has been shown to improve downstream model generalization across a variety of tasks, including image segmentation, classification, and detection [29,30,31,32,33,34]. Several approaches have been proposed for image synthesis with generative adversarial networks. In our specific application, data augmentation can enable better generalization over different environments or weather and lighting conditions, and it can also be used to balance our dataset or for sensor correcting, such as fixing noisy inputs and sensor modeling. The synthesis can be done in two- or three-dimensional spaces, in addition to spatio-temporal spaces such as videos. For example, Point Cloud GAN proposed a twofold modification to GAN for learning to generate point clouds [35]. Moreover, they provide a study for transforming images into point clouds. PrGAN [36] generates a distribution over 3D structures given 2D views of multiple objects taken from unknown viewpoints. On the other hand, Temporal GAN (TGAN) [37] generates videos using a temporal generator and an image generator learning a semantic representation of unlabeled videos. However, best results are still found in 2D Synthesis with state-of-the-art models such as StyleGAN [38], StyleGAN2 [39], and SWAGAN [40], which incorporate wavelets throughout its generator and discriminator architecture enforcing a frequency-aware latent representation, or StyleGAN extension for training with limited data using Adaptive Discriminator Augmentation (ADA) [41].

## 3. Dataset

**Figure 3:**
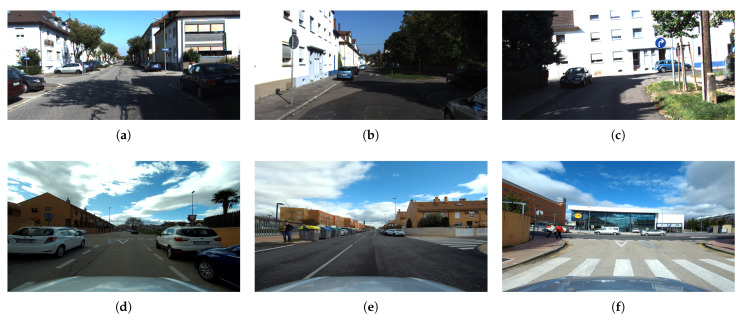
Some examples from the mid-sized city of Karlsruhe for the KITTI and KITTI-360 datasets and Alcalá de Henares (our dataset). The reader can appreciate the differences in the spatial distribution of street elements with respect to Figure 1, as well as the change in the field-of view between the KITTI cameras (**a**–**c**) and our dataset (**d**–**f**).

We addressed the intersection classification challenge by trying to exploit the most popular dataset available in the autonomous driving field, even though not explicitly designed for intersection detection purposes. Using these datasets would help us to easily compare our results from some of the previous works mentioned in Section 2. However, the first issue we faced was related to the locations where these datasets have been recorded. As can be seen in Figure 1, these datasets were collected inside huge metropolitan areas like San Francisco (Pandaset/Lyft5); Beijing; and other cities in China (ApolloScape/Baidu), Boston, and Singapore (nuScenes). The intersection visibility in these conditions is hampered due to several reasons: the width of the avenue and the related lens’ field of view, the separation of travel directions that make two-way traffic streets look like one-way ones, and the massive number of vehicles. One possible solution for the identification and classification in these extreme conditions would be, for example, exploiting the detection of traffic lights and the analysis of other car trajectories, such as in [16]. On the other hand, less urbanized areas like the ones in Figure 3 can benefit from the capabilities of classification networks. To the best of our knowledge, the only public dataset previously considered for the intersection classification problem in mid-sized cities is the KITTI dataset and its recent KITTI-360 extension. There are two issues that, after careful analysis, prevent state-of-the-art approaches based on image-based detectors from obtaining excellent results. First, as can be seen in Table 1, the number of intersection frames is relatively low. This makes the training phase of any network extremely challenging.

**Figure 4:**
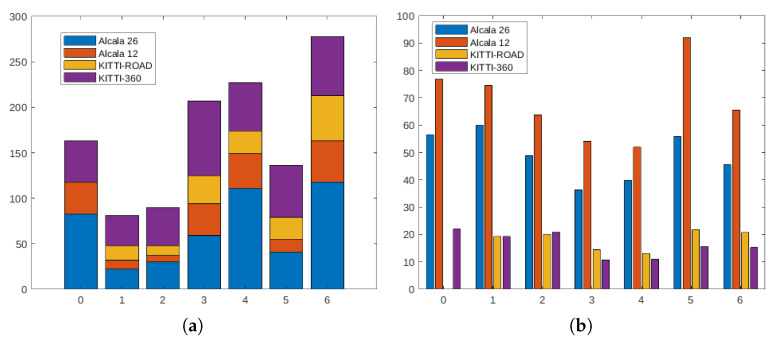
Distribution of intersection types by the seven considered road configurations (0–6) (**a**) and sequence length (**b**) for KITTI, KITTI-360, and the two sequences of our Alcalá de Henares dataset. As the reader may notice, the number of intersections introduced with our sequences doubled the number of intersections for every class. Moreover, the average frames associated with each of the intersections is also increased. KITTI is recorded at 10 Hz, while our sequences are recorded at 30 Hz.

**Figure 5:**
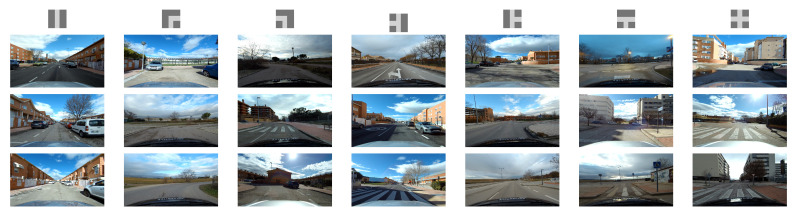
Each column shows three examples of the seven configuration geometries that were used in this work (with the *so-called* canonical images shown in the upper part of each column). The RGB examples come from our recorded dataset. We recorded the images at different times of the day using a different camera setup for more than 3:30 h of recording time, including more than 500 intersections.

Besides the frame availability, another interesting number to analyze is the actual number of intersections. As can be noticed in Figure 4, and considering only the numbers associated with KITTI-ROAD sequences (a subset of the original KITTI dataset), the number of per-type intersections is critical, far below the expected number of elements usually typically used for DNN training’s, especially for those networks that use sequences instead of the mere number of frames. Note that, for a given intersection, the visual appearance of the scene on consecutive frames has minimal changes. Together with the low frame availability, this forced us to pay special attention to the common dataset split phase: randomly choosing frames from the whole dataset was not an option, due to the multiple frames associated with every intersection. By randomly selecting frames, it would have been possible to include similar frames of the same intersection into both training and validation or testing, frustrating the separation efforts. As there is no easy solution for the availability issue, these considerations led us to create a much more extended dataset of intersections recorded in the surrounding area of Alcalá de Henares, Madrid, Spain, during the first half of 2021. Together with a set of scripts and additional descriptions, all the images will be released to the community and publicly available on http://invett.es/intersectiondataset. A current subset of the dataset, which contains all intersection geometries in different weather conditions and seasons, is used in this work, and a few images can be appreciated in Figure 5.

Among the datasets used in our work, one substantial difference lies in the availability of *single* vs. *stereo* camera head and *camera* (single-sensor) vs. *camera and LiDAR* (multi-sensor) configurations. This difference allowed or denied some of the configurations explained in Section 4.4.2.

## 4. Technical Approach

**Figure 6:**
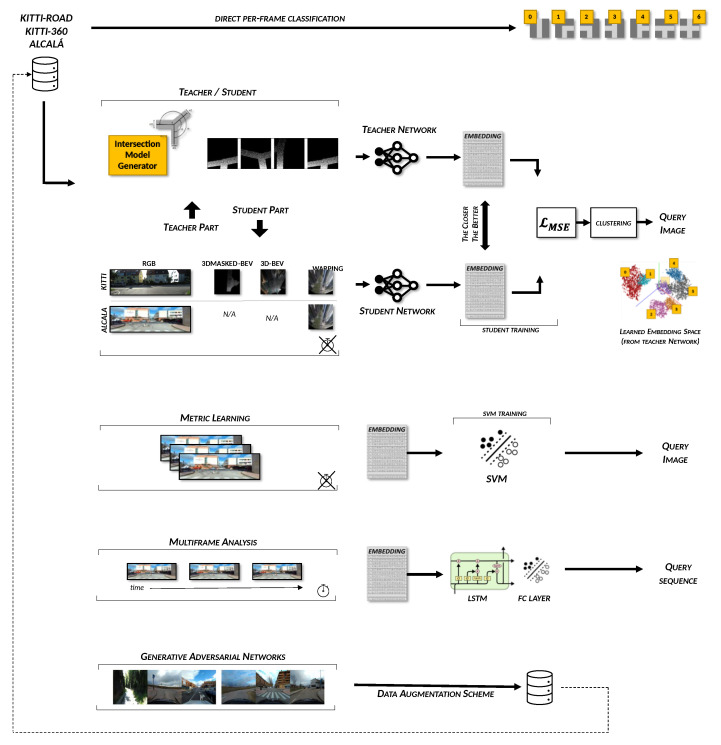
The picture includes all the approaches presented in this work, including Direct per-frame classification (Section 4.1), Teacher/Student (Section 4.4), Metric Learning (Section 4.2), and Multi-frame analysis (Section 4.5), together with the GAN-based data augmentation scheme. As the reader can notice, all the approaches except the direct per-frame classification involve using embedding vectors obtained from DNN systems, which in turn are further processed using the techniques described in the remainder of this work. The clock symbol indicates when a time-series analysis is involved.

This study’s purpose consists of the assessment of different methodologies addressed to detect the geometry of an upcoming intersection outside metropolitan areas. Toward this goal, we have evaluated the classification capabilities of state-of-the-art DNNs systems, including networks aimed at analyzing single-frame images as well as video sequences. Moreover, concerning the single-frame image analysis, we wanted to verify the performance gain that the metric learning and the Teacher/Student learning paradigm have with respect to a standard classification baseline. Different loss functions, including triplet-loss techniques, were also investigated. Each of these network configuration and training methods is carefully described in the following subsections, which explore our extensive research activity in detail. For the sake of simplicity, Table 2 and Figure 6 summarize all the presented approaches.

### 4.1. RGB Baseline

Our preliminary work [42] started our evaluations by assessing the end-to-end classification capabilities of two well-consolidated networks for image classification: ResNet [43] and VGG [44] networks. Throughout the subsequent research here presented, more network architectures have been incorporated to explore the capabilities of other backbones more intensively. In addition to all the common ResNet and VGG implementations, MobileNet-V3 [45] and Inception-V3 [46] architectures have also been investigated. The performances of each of the networks, as well as the evaluated loss functions, will be discussed in Section 5. Besides representing the more trivial learning paradigm, these end-to-end configurations allowed us to create an architecture baseline to compare each additional system. Following our first preliminary work in [42], regarding the KITTI and KITTI-360 sequences, we used the RGB images from the left camera of the stereo rig. For all the sequences of either KITTI or our datasets, in a similar way to what was proposed by other authors, we also prepared a second set of images containing 2D-homographies of the original images. Our intent is to create Bird’s Eye View (BEV) images for each of the correspondent RGBs, trying to help the classification network by eliminating cluttering elements of the scene, e.g., above horizon elements, while at the same time emphasizing the intersection geometry. Together with the images directly acquired from the camera, these two sets of images allowed us to perform a first classification assessment of the networks mentioned above and evaluate the improvements described in the following sections.

### 4.2. Metric Learning

**Figure 7:**
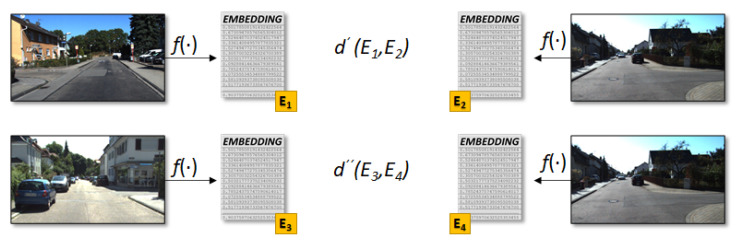
The distance between the two images is calculated using the numerical N-dimensional embeddings resulting from a generic DNN network. For example, considering the ResNet model, the resulting embedding $E_{x}$ dimension is 512. The intuition is that for different intersections the distance $d^{\prime }$ between embeddings is larger than the distance of same-class intersection $d^{\prime \prime }$.

The so-called *metric learning* represents an exciting and emerging technique in the machine learning community. This notation, used as a generic term to indicate a distance, a correspondence, or difference between elements, allows for a slightly different classification approach from the one presented in the previous section. As it is well described in [47], with a metric-learning technique, we can shift the classification problem towards an optimization problem so that pairwise or triplet-based distance constraints between items are both enforced and minimized using a specific loss function. From a technical perspective, the distance is evaluated starting from the *embedding vectors* associated with a couple of input images, where the embeddings are generated by a function f(·) in the form of DNN, see Figure 7. The idea is that given three images and the associated embedding vectors called *anchor (a)*, *positive (p)*, and *negative (n)*, where the anchor is the query point in the vector space, a generic distance *d* with respect to the positive and negative points in the vector space satisfies the Equation (1).

(1) $$\begin{array}{cc} d(f(a),f(p)) & <d(f(a),f(n)) \end{array}$$

In this work, we used two different implementations of the metric-learning technique depending on the specific problem we were analyzing, including an ad hoc implementation and the much more exhaustive work presented by Kevin Musgrave in [48] (please note that in the remainder of the manuscript we refer to this work as the metric library). This package allowed us to evaluate different distance functions apart from the generic $L^{2}$ distance, e.g., Cosine-Similarity and SNR-Distance [49]. Moreover, we used the concepts of *Triplet Margin Miner* to use specific positive-anchor and negative-anchor pairs. These concepts will become more apparent after the next section.

#### Triplet Scheme

Within the plethora of metric-learning different patterns, we used the *triplet* approach described in [50], where a set of three images $\left(M_{1}^{a},M_{2}^{s},M_{3}^{d}\right)$ belonging to the same domain, i.e., *M*, composed of one *anchor* class image $M_{i}^{A}$, a *same* class sample (positive-anchor) $M_{j}^{S}$, and a *different* class sample (negative-anchor) $M_{k}^{D}$, are passed through the triplet margin loss function. Again, the idea is to have a DNN generating a high-dimensional embedding vector associated with input images. The distance between vectors belonging to different intersection classes is higher than the distance derived from the vectors of same-class intersections. This loss function is defined similarly to each of the two parts of Equation (1), as follows:

(2) $$L=\sum_{i}{\left[d\left(f\left(M_{i}^{A}\right),f\left(M_{j}^{S}\right)\right)-d\left(f\left(M_{i}^{A}\right),f\left(M_{k}^{D}\right)\right)+m\right]}_{+}$$

where ${\left[\cdot \right]}_{+}$ means max(0,[·]) and $d\left(x_{i},y_{i}\right)={∥x_{i}-y_{i}∥}_{p}$ with *p* as the norm degree for pairwise distance and *m* is an extra scalar value used to extend the *margin* between the embeddings. As the reader might guess, not all the positive and negative triplets will have similar scores. Unexpected geometries might also generate confusion and lead to unsatisfactory results. For this reason, we proposed using the *Triplet Margin Miner* feature provided in [48], which allows us to specify a minimum distance threshold for the generation of the triplets.

### 4.3. RGB Metric

We began our investigation by directly verifying the benefits introduced by the metric learning approach. First, according to Equation (2), we evaluated different distance functions. Starting from the basic ${∥\cdot ∥}_{1}$ and ${∥\cdot ∥}_{2}$, we have expanded the distance function set to assess the impact of Signal-to-Noise Ratio and Cosine Similarity functions provided within the metric-library. As mentioned, Section 4.2, the metric library also offers the opportunity of using a *miner*. This solution allows us to collect those pairs or triples that exceed a preset threshold before calculating the loss function itself, working as a difficulty discriminator between the elements. Specifically, the *Triplet Margin Miner* has been used for this work, with two of its possible configurations: *“all”* and *“hard”*. The first of these configurations lets us calculate the loss value by using all the triplets that violate the preset margin. The second one allows for creating of a subset of the previous triplets, where the positive example is further away from the anchor than the negative example. These two configurations allow us to focus our training efforts on the most challenging examples for the neural network. However, for the sake of completeness, we also performed training without any miner, letting the system use any possible triplet regardless of its difficulty. As this training methodology works directly with distances, it is necessary to identify a metric to evaluate the performance of the system. The metric library provides different functions that can be used in this situation, such as Mean Average Precision@R (MAPR), Mean Average Precision (MAP), Precision-at-1 (PA1), and R-Precision (RP). All these values have been recorded during the training process to assess how the training was running. However, the value is taken into account to run the validation patience and select the best training is MAPR, as this metric operates directly on the embedding space and provides better information [51]. Once the model has been trained, it is necessary to find a way to use it, as an embedding does not directly refer to a specific intersection type. An easy and apparently trivial way could be to use the metrics above to calculate the minimum distance to a specific class representative vector. For example, using a clustering procedure, the representative vector might be the centroid of each cluster. However, apart from relying on such elements as centroids, we also evaluated the following methods. Following the distance concept, a first approach consists in using the Mahalanobis distance rather than the Euclidean distance to calculate the classification of each new example in such a way to capture better the distance with respect to the learned concepts. The second method consists of a transfer learning adaptation: once the network has been trained using the previous proposals, its weights are frozen, and a fully connected layer is trained to classify the embeddings returned by the network. Finally, the third proposal consists of training an SVM classifier using the embeddings of the training and validation sets. Once the classifier is trained, the implementation of the system in a real environment would consist of two steps: obtaining the embedding from the trained architecture and its classification employing the SVM.

All the training activities performed with this methodology, in addition to the three datasets already explained, have been carried out also with different data transformations which will be explained in detail in Section 4.4.2.

### 4.4. Teacher/Student and the Intersection Model

The second learning paradigm we wanted to evaluate with respect to the standard end-to-end scheme was the so-called *Teacher/Student*. Among all the possible applications, the idea behind this paradigm includes transferring knowledge from a simple domain to a much more complex one.

#### 4.4.1. The Intersection Model

**Figure 8:**
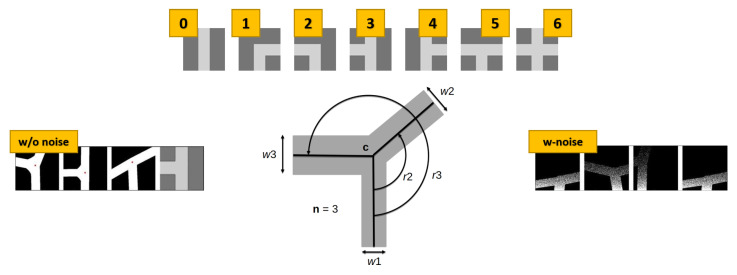
On the top of the image, the seven so-called *canonical* intersection classes along with the model used to generate the training dataset. In the leftmost part of the figure, identified with the label *“w/o noise”*, a triplet consisting of two samples of the canonical *type-three* (for simplicity also shown in the last box of the row) and a different one, e.g., *type-five*. Before passing these images to the teacher network during the training phase, random noise is added to each image by increasing extent along the vertical direction. The effect is shown in the rightmost part of the figure identified with the label *“w-noise”*.

**Figure 9:**
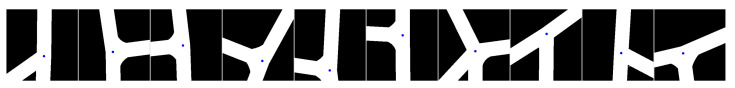
More examples of MBEV images generated with the model in Figure 8.

From a technical perspective, the starting domain from which we propose to transfer the knowledge consists of a synthetic set of simple Bird’s Eye View (BEV) images generated with the intersection generator proposed in [18,52]. This simple intersection generator, shown in Figure 8, generates all seven configuration classes commonly used in the literature in the form of binary masks, such as those shown in Figure 9. We refer to these images as Model-Based Bird’s Eye Views (MBEVs). These masks contain the shape of the most common intersections entities that can be found in mid-sized cities. The model parameterization allows us to generate different intersection geometries, i.e., changing the number and position of intersecting roads, the center position with respect to the generated image, and, finally, the carriageway of each road segment involved. Without pretending to be the definite intersection generator, as many configurations still cannot be represented with our proposals, e.g., complex intersections that can be typically seen in big metropolitan areas, this model allowed us to assess the intersection classification capabilities of the evaluated network configurations using the datasets proposed in Section 3. This model acted itself as a trivial data augmentation scheme for the CNN during the training phases of our teacher networks. To mimic the way in which some of the training images are created, we optionally added an increasing amount of random noise starting from the bottom part of the mask in a line-by-line fashion, as visible in Figure 8. More details will be provided in the following sessions.

#### 4.4.2. RGB Pre-Processing

To exploit the MBEVs images generated with the intersection model, we created a set of three different pipelines to transform the RGB images into a similar viewpoint. This set includes the following.

**Figure 10:**
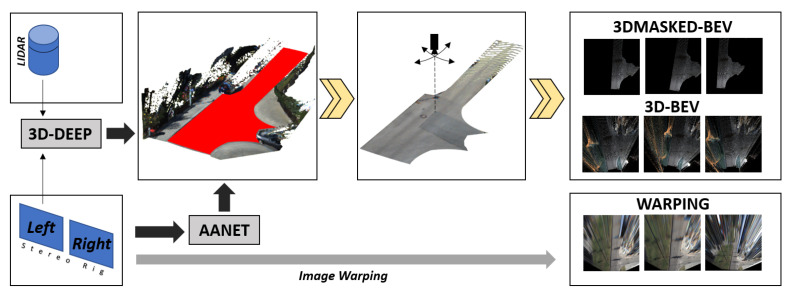
The figure depicts the overall pipeline used to generate the 2D planar images used in this work (apart from the original RGB-left camera). Among them, only the 3DMASKED-BEV uses the LiDAR data. The central part of the image shows the two methods of our *Data augmentation scheme*, i.e., the 3D-based realistic virtual camera that exploits the 3D point cloud-based reconstruction of the image (from AANET and 3D-Deep networks) and the 2D-based homography that usually generates substantial image distortions.

- 3D-Generated Bird’s Eye Views (3D-BEVs): this first transformation, applicable only to datasets that provides stereo camera configurations, creates a BEV representation of the scene using a 3D-reconstruction process; it is the most accurate 2D plan view that can be generated from images, as no distortions are introduced in this procedure. The effect of the virtual camera can be seen in the center box of Figure 10. We used the work in [53] to generate the depth image that, in turn, allowed us to create a 3D representation and then the desired 2D image. Please note that having a 3D representation allows us to change the virtual camera position retaining the scene’s consistency and simultaneously acting as a data augmentation methodology.
- Masked 3D-Generated Bird’s Eye Views (3DMASKED-BEVs): for this second transformation, we extended the previous pipeline by including the results in [54], to remove the 3D points that do not belong to the road surface and thus generate an image containing only road pixels. The main insight here is to evaluate whether the classification may benefit from less cluttered yet pre-segmented images. For this purpose, we combined the previous depth-image to the generated road-mask before creating the 2D view. The downside with this variation is that the approach in [54] only works with LiDAR data.
- Warping with Homographies (WARPINGs): this last transform tries to overcome the limitation of stereo and LiDARs availability at the cost of introducing distortions in the generated images. We applied standard computer vision techniques to create a homography between the RGB image and the desired 2D image. As homographies are only defined for flat surfaces, but common roads do always have at least slight deformations, this image transformation introduces distortions to the final image, as depicted in the *warping* images in Figure 10. Moreover, as we used fixed homographies, the actual attitude of the vehicle introduces similar distortion effects as the vehicle moves along its route. Nevertheless, as we have more than one frame-per-intersections in our videos, we can conceive this effect as a data augmentation scheme, as the same intersection visually appears different across successive frames. Please notice that this scheme is equivalent to the one proposed for the RGB-Baseline described in Section 4.1.

In addition to the basic RGB data, the three configurations were evaluated with respect to the learning schemes presented in our work. Please notice that 3D images are created only when LiDAR data is available.

#### 4.4.3. Applying the Teacher–Student Paradigm

The central insight besides the Teacher/Student paradigm is to learn a shared embedding space between the 2D images created with the intersection model and those generated by the aforementioned transformation pipelines. This approach is inspired by the work of Cattaneo et al. [55], which performs visual localization using 2D and 3D inputs in a bi-directional mode, teaching two networks to create a shared embedding space and thus enabling a two-way localization, starting either from 2D or 3D inputs. Recalling the metric technique described in the first part of Section 4.2, the teacher-student paradigm introduced some minor changes, in particular to Equation (1). In detail, given two instances of the same intersection class belonging to different domains, e.g., *Class 0* in domains *D1* and *D2* ($D_{C=0}^{1}$ and $D_{C=0}^{2}$) such as D1 is the space of the images with our intersection model and D2 is the RGB-transformed space, and two different non-linear functions f(·) and g(·) represented in the form of DNNs, the distance between the embeddings is lower than any other negative intersection instance, e.g., $D_{c=2}^{2}$. Formally, given the *Intersection-Model* domain *M* and the *Camera* domain *C* such that $M=C=\left\{0,1,2,3,4,5,6\right\}$, each of which contains the seven intersection typologies considered in our intersection model, and given one element $m_{i}\in M$, then Equation (3) is satisfied ∀i,j∈C|i≠j, where d(·) is a distance function.

(3) $$d\left(f\left(m_{i}\right),g\left(c_{i}\right)\right)<d\left(f\left(m_{i}\right),g\left(c_{j}\right)\right)$$

**Figure 11:**
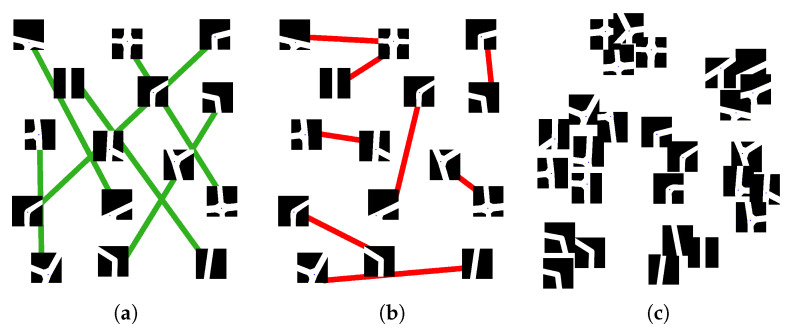
The picture depicts the position of MBEVs in the vector space together with their pairwise distances (colored edges), visually showing the intuition behind the metric learning approach. Same class (green) and inconsistent, different-class (red) edges in panels (**a**,**b**), respectively, are used to find a metric in a way that similar images are grouped together in clusters, as visible in (**c**). The images shown here are generated with the intersection model presented in Section 4.4. Besides this first intuition of constraints in terms of *pairs*, we can imagine *triplets* to further increase the complexity of the optimization problem, helping the system to obtain better and wider distances between clusters.

**Figure 12:**
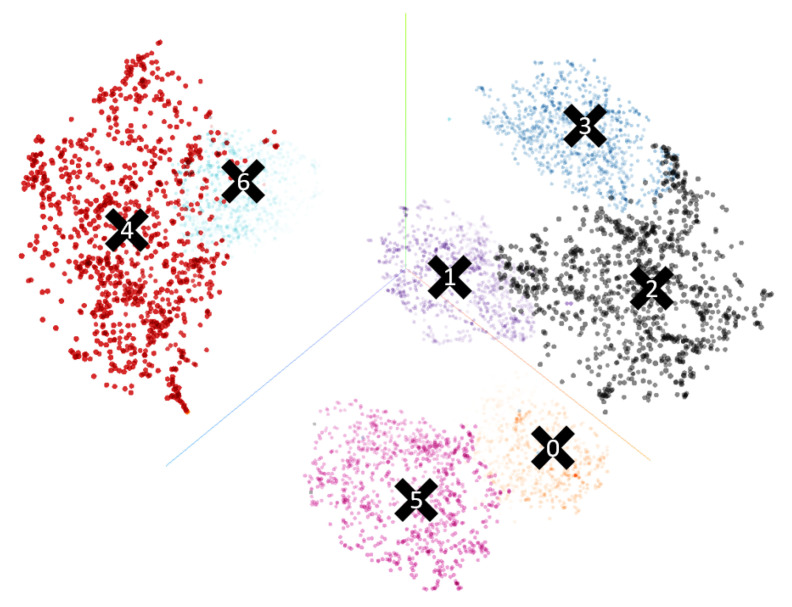
The embedding space is visually represented using 7k samples from the intersection model, using T-Distributed Stochastic Neighbor Embedding (t-SNE) function. Apart from the few outliers, the image depicts how the Teacher Network can separate the seven classes generated with our intersection model. In black, we conceptually represent the centroid of each of the clusters.

Regarding the teacher network, which is the first part to be trained, we trained it similarly as we have previously done with the RGB images, but this time we used the images generated from the intersection model, see Figure 11. Once the teacher model has been trained, we trained the student network using the preprocessed RGB images presented in Section 4.4.2 as input data in a way to obtain a similar embedding vector. Towards this goal, the loss-function of the student network is composed as follows:

(4) $$L=\sum_{i}\left[d\left(f\left(M_{i}^{A}\right),g\left(C_{i}^{S}\right)\right)\right]$$

where *M* and *C* are the model-domain and camera-domain, respectively, and Mean Squared Error (MSE) was used as distance function d(·) between the embeddings. Differently from Equation (2), here the triplet scheme is replaced by a pairwise distance between the embedding vectors. Note that to maintain a consistent distance within same-class classifications, $M_{i}^{A}$ elements were chosen not from the list of embedding vectors used in the training phase of the teacher network but rather from the average of 1000 new random samples generated after the teacher network was trained, i.e., never seen before from the DNNs. These per-class averages, i.e., cluster centroids, are shown in Figure 12 with black crosses and represent therefore our $M_{i}^{A}$ set.

#### 4.4.4. Training Details

A data augmentation process was introduced in both networks to avoid overfitting during the network’s training phase. We generated a set of 1000 per-class intersection configurations by sampling from our generative model for what concerns the teacher network. We applied a normal random noise to the seven *canonical* intersection configurations on each parameter involved in the intersection generation, e.g., width, angle, and intersection center, in a measure of 2.0 m, 0.4 rad, 9.0 m, respectively. For what concerns the noise, starting from the bottom of the image, we added an increasing number of random noises to each line to mimic the 3D density effect of 3DMASKED-BEVs. Regarding the student network, as the low number of intersections present in the two KITTI datasets in comparison with the overall number of frames, we performed data augmentation adding a 6-DoF displacement to a looking-down virtual camera initially set at 10.0 m and 22.5 m above the road surface and at 17.0 m and 22.0 m in front of the vehicle for the KITTI and KITTI-360 respectively. Due to the nature of type-1 and type-2 intersection classes, which contain any kind of curve without a specific curvature threshold, we zeroed the rotation along the vertical axis to limit the chance of assimilating these samples to the type-0 class. Our code leverages the PyTorch 1.6 learning framework [56], and both teacher and student images were scaled to images with size 224 × 224 pixels. At this time, despite its triviality, note that the point-density of 3DMASKED-BEVs is not constant over the distance with respect to the vehicle. Therefore, to simulate comparable MBEVs, we added a random noise proportional to the distance, see Figure 8.

### 4.5. Multi-Frame Analysis or Schemes

We continued our research by assessing the results of multi-frame learning schemes. As the system is intended for autonomous vehicles, it seems logical to think that the vehicle will get more information about the surrounding environment as it approaches the intersection, and the interpretation accuracies will increase as confirmed in [18]. Therefore, implementing a system that can exploit the spatio-temporal information from a sequence of images until the vehicle is inside the intersection should give even better results.

#### 4.5.1. Recurrent Neural Networks

With the idea of further refining the work done so far with the CNN networks, we investigated possible architectures that could be combined with them to perform temporal integration of the data. It seems logical to think that the system will be able to process more information and improve the results by temporal integration of the data. The two predominant models for this in state-of-the-art are the Gated Recurrent Unit (GRU) and LSTM architectures. These types of architectures typically have input packets of data vectors grouped into temporal sequences. As the CNN networks that have been used so far return embeddings, the only process necessary to group the two architectures lies in the temporal aggregation of the embeddings. For this, the following process has been followed. First, the images have been grouped into sets belonging to the same junction forming a single temporal sequence. These samples are then passed through an already trained CNN, which returns a sequence of embeddings. Each recurrent network was trained with the best-performing CNN previously trained for each dataset and data type configuration. Once the CNN has processed all the batch sequences, the shortest ones are zero-padded so that all the sequences that compose the data block supplied to the recurrent network have the same length. Finally, as both GRU and LSTM return feature vectors, it is necessary to classify them. Similarly, as has been done for the metric approaches, we used two different methodologies: the inclusion of a fully connected layer trained simultaneously with the recurrent architecture and the use of an SVM classifier in a similar way as described in Section 4.3. With both architectures, we made several architecture variations to perform an adequate ablation study. These variations range from the number of layers in the network or the hidden layer’s size to the internal dropout of the architecture. The training has been performed using all the available datasets and respective transformations presented in Section 4.4.2. More detailed information on training will be provided later in Section 5.4.

#### 4.5.2. Video Classification Networks

Apart from the recurrent architectures, a second exciting network design for temporal sequence analysis is represented by the expansion of standard 2D architectures. The basic idea is to feed a standard classification network with a set of consecutive images, allowing for the creation of so-called spatio-temporal filters. The main issue with this architecture is the huge increase in terms of network parameters with respect to the corresponding 2D network version, which makes the training phase harder and computationally demanding. Moreover, the 3D extension also seems to neutralize the benefits of pretrained models [57]. Extensions to these basic video recognition architectures were introduced in the very last months by the works presented in [58], where a family of efficient video networks is presented. A preliminary remark that must be made is that all of these architectures are usually employed for the detection of specific activities in video sequences. Typical datasets include KINETICS [59], Charades [60], EPIC-KITCHENS [61], or something-something [62], where a huge set of activities is provided in terms of short videos. However, it is our opinion that similar actions or high-level *concepts* could also appear in a sequence representing a vehicle approaching a type of intersection. With the idea of having a similarity between the action *“picking something up”* and *“approaching a type-x intersection”*, and the promising results of these approaches, we decide to perform a set of preliminary experiments using the works proposed in [63] and generally contained within the PyTorchVideo framework. To the best of our knowledge, this is the first time this kind of approach is evaluated in the intersection classification context.

### 4.6. Artificial Data-Augmentation: GAN

**Figure 13:**
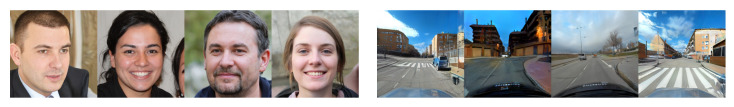
On the left, an example of the performances of StyleGAN-2. On the right, an example of the output of the StyleGAN-2-ADA network after 800k epochs. The number of face images in the FFHQ [38] dataset is impressively high in comparison with the few sequences of our combined sequences. For this training, we used all the images available in both Alcalá and KITTI sequences, without any split. However, to avoid presenting too similar images, we decimated the input images to have a constant frame rate equal to 1 fps.

As previously stated, one of the major issues that affects all the aforementioned techniques is the limited availability of intersections, primarily in terms of intersection instances rather than with regard to the number of frames. In an attempt to overcome this limitation, we implemented and compared several GANs frameworks, from more simplistic approaches like DCGAN, WGAN, and CGAN to the last state-of-the-art networks, such as StyleGAN2, SWAGAN, or StyleGAN-ADA, which includes an adaptive augmentation procedure to deal with limited data regimes [41]. Given the limited amount of data, the best results in terms of quality and regularization metrics were founded for SWAGAN and StyleGAN2 with the adaptive discriminator augmentation. SWAGAN [40] noticeably increases computational performance; however, some checkboard artifacts appeared in the images, and it achieved worse perceptual path length regularization. Therefore, we finally opt for StyleGAN2-ADA. Figure 13 depicts some generated examples of the latter after 800k epochs.

**Figure 14:**
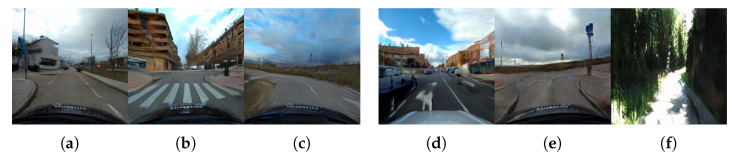
Some examples of good generated RGB intersections, included in the new augmented dataset (**a**–**c**), and discarded ones (**d**–**f**).

Following the intuition that feeding the network with warped images could improve the generative process, as the network would be able to focus only on intersections, we performed different tests training the GAN with RGB images and with warped images. In order to balance less represented classes, we trained the GAN following a conditional setting. However, for RGB images, the best results were found for the non-conditional framework. Therefore, we create the new augmented dataset generating 10,000 random images and labeled them frame by frame, discarding those which do not depict a clear intersection. Figure 14 shows some examples of good and bad generated images. One issue we found with the generated images is that most of them were similar to the original ones. This is probably because the number of training images obtained with all the sequences of both KITTI and Alcalá dataset is more than six times smaller than the 30k training images used in the StyleGAN-ADA original contribution [41]. Notwithstanding, results show better classification performances with the augmented dataset.

**Figure 15:**
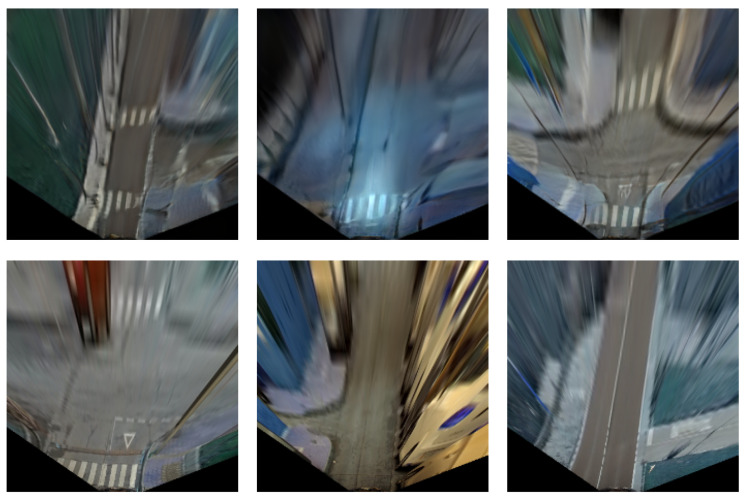
In the first row: some examples of generated images when training the GAN with warped real images. In the second row: examples of real warped images.

Although most generated images were realistic enough, i.e., showing at first sight a clear road environment, we had to discard ~82% of the RGB images due to inconsistencies on the road or an unclear intersection. We are currently tackling this issue by introducing new elements to the standard StyleGAN proposal, and we plan to present the outcomes in future work. On the other side, generated warped images showed similar performance when training in a conditional setting, probably due to the amount of information contained in these types of images. The latent space is better regularized, and generated images are closer to the real ones in the embedded space. Therefore, we generate 10,000 warped images and added them to the warped training set. Figure 15 shows some examples of the generated warped images under a conditional setting. It can be seen that they are realistic when compared to the real ones.

## 5. Experimental Results

The following section presents the extensive experimental results of all the tests we have performed with the different methodologies. These tests have been performed using a NVIDIA DGX-A100 server. The test battery, as described below, has been configured using Weights & Biases [64], which has allowed us to perform an extensive ablation study using the automatic search procedure called *sweep*. By performing a sweep, the Weights & Biases service allows for automatically searching the best combination of hyperparameter values. In order to work with embeddings of similar size, and given that different network architectures have been used, a space reduction should be made in those architectures that return larger feature vectors. To make such an adaptation, a fully connected layer has been used to reduce the size of the embeddings to 512 values. The selected architectures are ResNet and VGG in all its versions, the two versions of MobileNet-V3 and Inception-V3. The test datasets have been divided, regardless of the number of images contained in each dataset, into the following percentages: 70% for the training set, 20% for the validation set, and 10% for the test set. To compare the improvements provided by the proposed methodologies, a sweep has been initially configured to find the best training solution for the proposed networks by direct classification of the RGB and WARPED images. The results obtained can be seen in Table 3, which will be used later in the comparisons. To encourage the research and development in the context of intersection classification as well to allow future researchers to compare their work with respect to ours, our code is available in our institutional repository on https://github.com/invett/nn.based.intersection.classficator, Access Date: 11 September 2021.

### 5.1. Teacher Training

The *teacher* is trained with artificial images created at run-time. These images try to simulate intersections as they were obtained from a Bird’s Eye View, to create an easy yet effective classification problem for the *teacher* network. As the primary use of the *teacher* network is to obtain a set of embeddings that can represent road junctions useful for the training of the *student* network, the loss function used is Triplet loss. As in the previous work, this decision was taken to separate as much as possible the space between the embeddings of different types of intersections so that two similar types are as close as possible and two different types as far apart as possible. Because the images are generated at run-time, once the anchor and the positive samples are chosen, the negative one is selected randomly from the remaining labels. We evaluated the performances of the teacher network using all the proposed architectures as a backbone and a set of 2000 artificial images for training and 1000 for validation. All training runs, regardless of the model, quickly became overfitted. The accuracy values in the training and validation sets reached 100%, so the model stopped learning and was no longer generalized. In case the architecture was too deep, the training was repeated with a customized network, which contained only one or two convolutional blocks (Conv + BN + ReLU) depending on the chosen configuration and a classifier composed of a fully connected layer. The results obtained were similar, so the latter architecture was discarded. In our opinion, this overfitting may be due to the use of the synthetic images created by the crossover generator as those images are effortless to classify. Therefore, the strategy to follow, as we believed that the ease with which the synthetic images were clustered in embeddings was going to be of great help in the training of the student network, was to select as the trained network the checkpoint immediately before the moment in which the precision value in the validation set reached 1. As we can see in Figure 12, performing a clustering analysis over the resulting set of embeddings clearly shows how the CNN can distinguish our intersection classes. As we want to use this information to train the student network, the centroids of each cluster are calculated to be used as reference marks in the subsequent training.

### 5.2. Student Training

In order to explore the widest solution space possible, a Weights & Biases [64] sweep has been deployed with the following parameters, randomly chosen at each iteration:

- Learning Rate: max: 0.01 min: 2.5 ×$10^{-6}$
- Optimizer: adamW, adam, rmsprop, sgd, ASGD, Adamax
- Loss Function: SmoothL1, L1, MSE
- Batch Size: 8, 16, 34, 64, 128

The last two transformations have not been applied to the Alcalá sequences as they do not have stereo or LiDAR information. For more detailed information on datasets and data transformations, see Section 3 and Section 4.4.2. In Table 4, we present the results obtained using different input data types (RGB, Warping, 3D-Images and 3D-Masked) for the three used datasets (KITTI-ROAD, KITTI-360, and ALCALÁ). As the training activities have been carried out with Weights & Biases sweeps, the table reports the maximum accuracy values for each architecture. Regarding the testing phases, we reported the value corresponding to the best performance obtained from all the architectures for each input data type. Our experimental activities were addressed to verify:

1. first, how the different data transformations exposed in Section 4.4.2 affect the results in the Teacher/Student paradigm and the possible benefits;
2. second, whether the use of the Teacher/Student paradigm substantially improves results over the direct classification of the data;
3. third, if the data recording methodology and the camera angle are critical points in obtaining good results; and
4. fourth, whether the inclusion of new architectures in training produces a significant variation in the results obtained.

**Figure 16:**
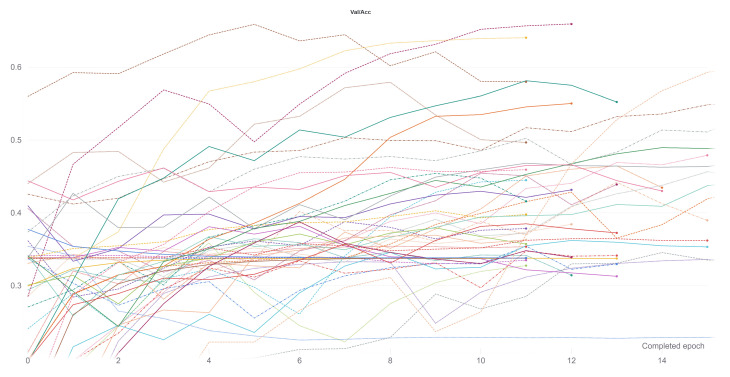
Example of a graph of the training results of the first 100 runs of a Weights & Biases sweep. The plot shows on the ordinates axis the Validation Set, Accuracy metric used to evaluate the performances of our system. As the reader will notice, slightly different parameters lead to considerable differences in the accuracy metric values.

If we look at the results in Table 4, we can see no substantial difference in the precision values according to the type of input data. If we take, for example, the results in the KITTI-360 dataset, the lowest validation value is 0.79 and the highest 0.86, a difference of 0.07. The differences in test values are equally minimal, 0.08 between the value achieved with the 3D-Masked data and the value achieved with the RGB images. This pattern seems to be repeated in all the datasets, which leads us to think that the preprocessing of the data does not seem to be such an important issue. If we look at the validation results obtained by the different architectures, there does seem to be a notable difference. For example, in the RGB images of the KITTI-ROAD dataset, there is a 0.25 difference between best and worst architecture. However, although similar differences are repeated between the different datasets and data types, the best- and worst-performing architectures do not seem constant. This variation leads us to think that other factors cause the differences and not the selected architecture. Regarding our second working hypothesis, the results do not seem to be very enlightening. If we compare the results obtained respectively with the baseline and the Teacher/Student approaches (see Table 3 and Table 4), the differences between the values achieved on validation are minimal, of the order of 0.01. The accuracy values achieved in testing are also quite similar, the highest difference being 0.11, in the training of the KITTI-ROAD dataset with RGB images. The results obtained in our previous work showed a slight improvement in almost all the Teacher/Student paradigm results concerning the direct classification. However, this improvement does not seem to have appeared during the process of the current research. We believe that this is because using the Teacher/Student paradigm is a risky option, and the results vary considerably depending on the initial configuration of the problem, especially if the data available are not extensive. Finally, to validate our third working hypothesis, we compared the results obtained using the KITTI dataset and both the Baseline and the Teacher/Student approaches (see Table 3 and Table 4). As can be seen, the results in the Alcalá dataset, both in direct classification and with the Teacher/Student methodology, are substantially higher than those achieved in KITTI in the same category. A clear example of this is the difference in accuracy between the KITTI-ROAD dataset and the Alcalá dataset when classifying RGB images. In direct classification, the validation value for the Alcalá dataset is one and a half decimal places better than in KITTI-ROAD, and the test value is almost four decimal places higher. These results are repeated within the Teacher/Student paradigm, with the Alcalá dataset being one and a half decimal places better on validation and three decimal places better on testing. These results, in our opinion, are very enlightening. The two KITTI datasets suffer from two things: lack of images and field of view. These two points have been addressed in the Alcalá dataset. We believe that the evident improvement of the results is mainly because the Alcalá dataset has a much wider field of view than the KITTI datasets. After all, although it also has more images, the difference is not so significant, especially with the dataset KITTI-360 (see Section 3). As the Alcalá dataset is currently a proof of concept, a significant improvement in the image number and field of view will be addressed with a complete dataset that will be available in the next months on http://invett.es/intersectiondataset. In addition, the lack of images in the KITTI dataset also seems to lead to some instability and overfitting in the training that we have observed during Weights & Biases sweeps, see Figure 16. This leads us to think that perhaps the conclusions obtained on our first two hypotheses are not definitive as it would be necessary to work with a much more complete dataset.

### 5.3. Metric Learning Training Phase

Due to the results obtained with the Teacher/Student paradigm, and the conclusions we have drawn from them, we believe it is necessary to use the data available to us more extensively to find better results. That idea leads us to the metric learning library [48]. The following process was used in the Teacher/Student methodology to calculate losses when training the student network. First, within each batch, the distance between each student’s network embedding and the centroid of the corresponding label is calculated. Then, losses are calculated based on how close the embedding is to the corresponding centroid according to that measure. Finally, losses are balanced according to the weights of each class and averaged. Thus, if the batch has eight data samples, losses will be calculated as a function of eight different distances. Unlike the Teacher/Student methodology, the metric library does not use a teacher to set the reference centroids to each label but uses distance functions to separate/join as much as possible the embeddings returned by the network according to their labeling. This methodology change allows the number of distances computed per batch to be substantially increased as each batch element’s distances can be calculated. The actual number of distances to be estimated per batch is given by nk, where *k* is the number of batch elements needed to determine the distance and *n* is the batch size. This exhaustive way of calculating losses during the training process, according to our criteria, could somehow circumvent the lack of data in the training sets. Our experimental activities were addressed to verify:

1. first, whether the new comprehensive way of calculating batch losses helps to alleviate the problems detected in the previous paradigm with the lack of images and
2. second, once the lack of images is solved, the usefulness of the data transformations and the improvement over the direct classification can be verified again.

Before discussing the results, we should comment on specific points that differentiate them from those shown in the previous paradigm. As the triplet loss function is implemented within the library and worked quite well in the teacher network training, it is the one that has been chosen to perform all subsequent Weights & Biases training sweeps. In addition, the implementation of this loss function allows selecting between different functions to calculate the distance between embeddings, which allows a broader field of exploration. In order to explore the widest solution space possible, as in the previous methodology, a Weights & Biases [64] sweep has been deployed with the following parameters, randomly chosen at each iteration:

- Learning Rate: max: 0.01 min: 2.5 × 10${}^{-6}$
- Optimizer: adamW, adam, rmsprop, sgd, ASGD, Adamax
- Distance Function: SNR, Cosine Distance, Pairwise
- Batch Size: 8, 16, 34, 64, 128
- Margin: max: 5.0 min: 0.5 q: 0.5
- Miner: All, Hard

**Figure 17:**
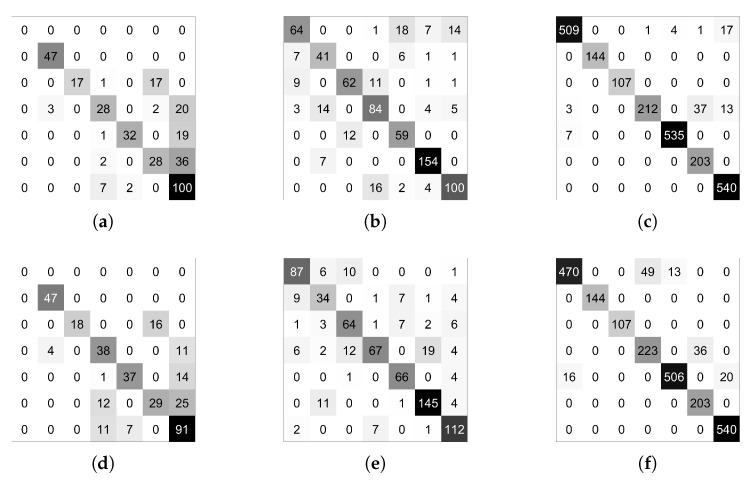
Different confusion matrix results in (**a**) student KITTI-ROAD, (**b**) student KITTI-360, (**c**) student Alcalá, (**d**) metric KITTI-ROAD, (**e**) metric KITTI-360, and (**f**) metric Alcalá. Please notice that in panels (**a**,**d**) we did not introduce straight roads (type-zero intersections).

As a comment on the above parameters, the margin is a parameter of the Triplet Margin Loss that can be stated as the desired difference between the anchor-positive distance and the anchor-negative distance. The Metric library offers different options to calculate the model’s accuracy from the embeddings returned by the network. During the training, several of them have been registered in order to have a global view of the training, but among all of them, MAPR [51] has been selected to establish which is the best performing model because we believe that it can be the one that best represents a good separation between classes. As we stated in Section 4.2, as MAPR is not reasonably comparable to accuracy and it is necessary to establish a direct classification methodology, the three proposed methodologies have been tested to obtain the results. Because these methodologies have only been used for testing, the validation values of the training cannot be fairly compared with other methodologies such as Teacher/Student or direct classification. Initially, the training of a fully connected layer to classify the embeddings returned by the architecture was used as a testing methodology. This methodology was discarded since the validation values of the training were relatively low, especially compared to the other methodologies, and therefore the testing values were expected to be even lower. Once this first methodology was discarded, we tested the following two methodologies, SVM and Mahalanobis distance. The results of both methodologies are similar; however, in most cases, the SVM was slightly better, so given the substantially longer computation time required to calculate the covariance matrix to use the Mahalanobis distance, it was decided to use only SVM to perform the tests. Please notice that the values for the Metric Learning paradigm reported in Table 5 always belong to the value returned by this last methodology. If we look at the testing results between Teacher/Student and Metric learning paradigms, see Table 4 and Table 5, we can observe that the higher number of comparison examples per batch has not substantially improved accuracy. Many values are similar or differ by a few decimal places, such as the warped images in the KITTI-ROAD dataset or the RGB images in the KITTI-360 dataset. In some cases, as with the 3D images in the KITTI-ROAD dataset, it has even worsened slightly. Therefore, we believe that the same problem of missing images can be seen in both methodologies and that further comparison between the available data has not led to a substantial improvement. In turn, the comparison between the Baseline and the Teacher/Student approach (Table 3 and Table 4) yield similar conclusions to those in Section 5.2 because, as mentioned above, the values are still very similar. Focusing on the previous comparisons with the Teacher/Student paradigm, the training using this new methodology seems to validate the assumptions shown in the previous section. If we compare the results obtained in the Alcalá dataset with those obtained in the other two KITTI datasets, we can observe a substantial improvement in accuracy values. The accuracy reaches its highest level, 0.3 higher, compared to the Alcalá dataset and the KITTI-ROAD dataset using RGB images. These results lead us to think that the critical point, as in the previous methodology, is above all the field of view of the camera with which the images are recorded. Figure 17 shows the confusion matrices of the best tests by training methodology and dataset. As can be seen, the results are pretty good, more than it might seem if only the combined accuracy of the seven classes is evaluated. These disaggregated results show that the system’s overall performance is good and that with favourable starting conditions, such as a better dataset, it can be excellent.

### 5.4. Multi-Frame Scheme Results

It seems a reasonably straightforward deduction that vehicle traffic is an event that runs over time. The approach to an intersection is not an event that only exists at point *t*. As the vehicle progresses to the junction, it seems logical to think it will receive more information. Therefore, it appears that if we can group all the information from instant *t* to instant t+n when the car is already at the intersection, we should achieve greater accuracy when classifying each intersection. With this idea in mind, the next step we have considered is to group, in order of approach, all the embeddings that belong to the same intersection to be classified as a *single sequence*. Our experimental activities were addressed to verify

1. first, we check if the temporal integration helps achieving our goal of classifying intersections and
2. second evaluate whether this new approach offers more light on the previous results.

As explained in Section 4.5.1, the inclusion of an RNN was the most straightforward methodology to implement as it can directly work with embeddings and integrate them. Training has been performed with two selected RNN architectures: GRU and LSTM. Preliminary results for both structures were very similar with those belonging to the LSTM network being slightly superior. Therefore, the subsequent training was performed with a LSTM architecture. Tests have also been performed in such a way that it is possible to obtain a final classification of the embeddings returned by the RNN, being the options to choose, as specified in Section 4.5.1, SVM, Mahalanobis, and Fully connected. In this case, unlike in the single-frame approaches, the best results have been obtained by training a fully connected layer while training the RNN network architecture. In order to explore the most comprehensive solution space possible, as in the single-frame approaches, a Weights & Biases [64] sweep has been deployed with the following parameters, randomly chosen at each iteration:

- Learning Rate: max: 0.01 min: 2.5 × 10${}^{-6}$
- Optimizer: adamW, adam, rmsprop, sgd, ASGD, Adamax
- Loss Function: Cross Entropy, Focal
- Batch Size: 16, 34, 64
- FC dropout: max: 0.5 min: 0.1 q: 0.1
- LSTM dropout: max: 0.5 min: 0.1 q: 0.1
- LSTM hidden layer: 256, 128, 64, 32, 16, 8
- LSTM layers: 1, 2

As a comment to the above parameters, the dropout of the fully connected layer is located just between the embedding and the classifier. The Focal loss function has been implemented from [65]. Regarding our first assumption, looking at Table 6, it does not seem that temporal integration has significantly changed the results. The validation values have increased in some cases, as in the KITTI-ROAD 3D images, but the test value is significantly worse, indicating overfitting. However, if we look at the results in the Alcalá dataset, the values for the two available paradigms are relatively similar. This connection leads us to think that the problems from previous approaches have been transferred to this one. Some comparisons were made between the results obtained by the LSTM and the results obtained by the single-frame approach. A simple temporal integration was made by a voting process between the results of all the frames of the same sequence to make a fair analysis. The results were very similar and analyzing them in-depth leads us to confirm the assumptions in previous points. The problem lies on two fronts: the number of images and the viewing angle. The lack of images means that when grouping them in sequences, the examples used to train the LSTM are smaller than in a single-frame approach, so it seems logical that the overfitting has increased. The lack of viewing angle in the KITTI datasets means that the network does not have enough information to extract good features from the crossing, something that cannot be solved with temporal information. If the feature extractor cannot divide the embeddings correctly, the LSTM will not be able to classify accurately.

### 5.5. GAN Related Results

We generated 10,000 RGB images and 10,000 warped images. As stated in Section 4.6, we use a conditional setting for the warped images but a normal one for the RGB images, as we noticed an important detriment in performance. Figure 14 and Figure 15 show some examples of RGB and warped generated images, respectively. Before including the artificially generated images in our original train dataset, we tested if they were close to the real ones by feeding them to our model trained with images of KITTI-ROAD, KITTI-360, and our datasets, and comparing the codes in the embedding space. In order to do so, we calculated the distances of these codes to the nearest clusters’ centroids previously computed with the real images. We did the same with a random dataset and checked the statistics of the three setups, showing consistency among the generated and real images, while random images’ embeddings were far from all centroids. Table 7 shows accuracy results for the Teacher/Student paradigm as well as for metric learning scheme for both RGB and warped images. Please note that we use MAP metric for validation results in the metric learning scheme. Dataset *ALL* refers to KITTI-ROAD, KITTI-360, Alcalá-1, and Alcalá-2, while *ALL AUGMENTED* refers to the same datasets together with the generated images. An improvement in test performance can be seen for the four setups when augmenting the dataset with GAN images. In the RGB setting, validation accuracy is lower in the case of the augmented dataset, which reaffirms our hypothesis that it improves generalizability.

### 5.6. Pytorchvideo

Our pilot experiments have been performed using ResNet3D and X3D [58] networks, together with the KITTI-360 and Alcalá datasets. Few changes to the original code were applied. Among them, we removed all the mirroring data augmentation schemes in the Charades dataloader implementation and modified the number of classes from 157 to our 7 basic geometries. Another distinctive feature of our experiments is related to the length of the input data, which strongly differs from the original video feed. As the intersections’ frames were manually selected, we modified the part of the code where a clip is randomly sampled from a whole video sequence. Our experimental activities were addressed to verify the following:

1. First, how the video-analysis networks handle both RGB and MBEVs images, comparing their performances with respect to the learning schemes proposed in this research. This test was executed using the KITTI360 and KITTI360-masked imagery.
2. Second, how different frame rates affect the classification capabilities. For this second test, we used the Alcalá-1 sequence, subsampling the original 30 fps to 6 and 15 fps. These experiments could not have been performed with the KITTI sequences due to the dataset low frame rate. Please notice that all the *actions* datasets originally used with these networks share the same length, which is not realistic with videos containing intersection approaches due to different speeds of the vehicle while approaching a generic intersection.

**Figure 18:**
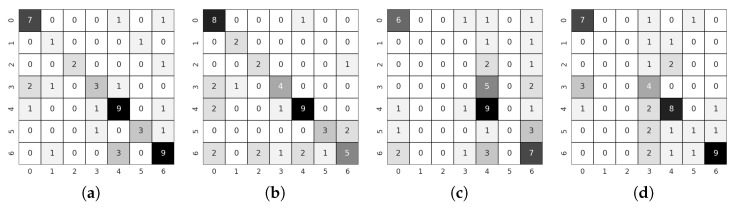
Different frame length test. (**a**,**b**) ResNet3D model with input at 15 or 6 fps. (**c**,**d**) X3D model with input at 15 or 6 fps. Dataset: Alcalá-1, test-split, 51 total intersections.

**Figure 19:**
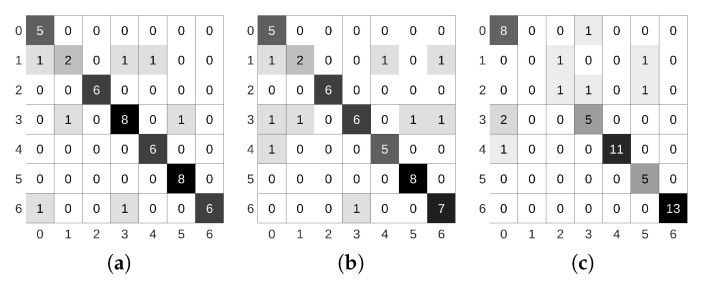
LSTM results using KITTI360 dataset and RGB data (**a**), MBEVs (**b**), and Alcalá-1 RGB dataset (**c**).

**Figure 20:**
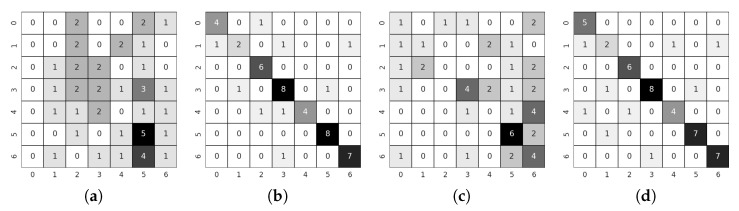
Different view test. (**a**,**b**): ResNet3D model, using RGB and MBEVs, respectively. (**c**,**d**): X3D model, using RGB and MBEVs, respectively. Dataset: KITTI360, test-split, 48 total intersections.

We repeated each of these tests using both the aforementioned ResNet3D and X3D approaches. We trained the system on a NVIDIA DGX-A100 server, following the video classification tutorial provided in the PytorchVideo GitHub repository, a variable epoch number between 200 and 1000 iterations with the CosineAnnealingLR Pytorch scheduler and the AdamW optimizer with different learning rates between 1 × 10${}^{-1}$ and 1 × 10${}^{-5}$ and weight decay set to 1 × 10${}^{-4}$. As for the image size, similarly to all the other approaches, we used a standard 224 × 244 size. Regarding the first experiment, tests showed us that different clip frame rates do not have a significant impact on the prediction performances, see Figure 18. This is of particular interest and suggests that high frame rates are not strictly required for this analysis, suggesting that the network can infer the geometry with just a few frames. A first interesting comparison can be made with respect to the different frame length experiment. As shown in Figure 19c, the performances of the LSTM system are better than all the different experiments performed with both the ResNet3D and the X3D models and the different time lengths used. Note that the Pytorch implementation of the LSTM considers different frame lengths, something that by its nature PytorchVideo framework does not. This is a clear advantage in favor of LSTMs approaches, as the approach to an intersection trivially depends on the vehicle’s speed. This issue might be solved with approaches that involve multiple pathways for activity recognition, such as the SlowFast [66] approach. However, due to technical issues of the PytorchVideo code, which is nowadays in its infancy, we were unable to further investigate this model. Last, given the results, we can conclude that the investigated networks do not achieve better results with respect to the RGB inputs. The second experiment further corroborates the latter opinion, and here the advantages of MBEV images are undisputed. As shown in Figure 19a–d, both the transformed images have better results with respect to the corresponding RGB images. Regarding the comparison between ResNet3D and X3D architectures, no clear advantage within the two architectures has been observed. The comparison with respect to the most similar temporal sequence-oriented architectures is shown in Figure 19. As the reader can see from Figure 19a, the LSTM approach outperforms both ResNet3D and X3D approaches of Figure 19a,c, while the corresponding MBEV experiments obtain much more similar results, see Figure 19b vs. Figure 20b,d, respectively, for ResNet3D and X3D.

## 6. Conclusions

This work presented an in-depth study of the intersection classification problem. Toward this goal, we explored the capabilities of state-of-the-art neural network approaches for image classification, considering different approaches such as single-frame, video-analysis and 3D-networks, non-traditional learning techniques for the specific intersection analysis subject, as well as different data source, e.g., RGB cameras and LiDARs sensors. An extensive experimental activity has been performed to validate each considered approach and its contribution towards the intersection classification problem. As a conclusion to this research work, we can obtain the following statements. First, as also mentioned in [21], is the critical importance of the dataset. As seen throughout the presented results, the number of available intersections plays a crucial role with all the tested approaches, achieving better results with the dataset with more intersection sequences. Despite the assertion’s simplicity, we claim that a complete analysis of the issue still requires a much broader set of intersections. Past state-of-the-art approaches limited to ad hoc datasets or the KITTI dataset series represent an essential yet limited contribution to the whole problem. For this reason we plan to release our recorded intersection dataset together with the *per-frame* and *per-sequence* annotations. Our contribution will provide a meta dataset containing not only our multi-season sequences but also meaningful annotations of current state-of-the-art datasets. Nevertheless, even with the aforementioned dataset at its present state, this research allowed us to prove functional requirements for the scope. Among these, the angle of view of the camera with which it was recorded is critical. Results show that detecting the intersection needs as much information as possible about what stands in front of the vehicle. In such a case, with limited environmental information from the imagery, e.g., with the KITTI imagery as discussed in Section 5.4 and Table 6, simply integrating temporal data in the form of the output of CNN networks can frustrate all the efforts and benefits offered by RNN variants. On the other hand, the results do not seem conclusive when using other training paradigms such as Teacher/Student or metric learning, or temporal integration. The results show no significant difference between these paradigms and direct classification. However, based on our experience, we believe that this is due to the limitations of the datasets. Therefore, it is possible that if these tests were repeated on a larger dataset, the results could be much more decisive than those obtained during this research. Same conclusions can be drawn from the preprocessing of the data, e.g., Bird’s Eye View. Currently, there is no conclusive evidence that image transformations represent a benefit or a detriment towards the intersection classification process. Regarding the scarcity of training and testing data, this work presented two different contributions. First, we presented a preliminary dataset of intersections recorded in the surrounding area of the Universidad de Alcalá. This cross-season dataset will be published in the following months, and we consider it will set an important milestone in the context of the intersection classification. Second, we prepared a new artificial set of intersections implemented using a Generative Adversarial Network approach which showed an overall improvement in the results. To the best of our knowledge, this was the first time that this approach has been tested in the context of intersection classification. We believe that combining authentic images with artificial images would be ideal, given the ease of obtaining the latter and as a means to balance the dataset. This second research line is of great interest, and we plan to write a second paper focusing only on this line of work. First, we want to explore the regularization of the latent space in order to achieve controllability in the generation process beyond the type of intersection. This would allow us to gain insights about the inner workings of our classification network and shed some light on which are the most essential features for achieving good performance. Second, by introducing our network in the GAN training loop, we expect to achieve more realistic intersection images, where the generative network focuses on what is most important for our problem.

## Figures and Tables

**Table 1:** Intersections frames per-class, all datasets.

Sequence	0	1	2	3	4	5	6
Alcalá-1 (jan26)	4693	1321	1467	2144	4429	2291	5374
Alcalá-2 (feb12)	2686	745	446	1893	1973	1287	2948
KITTI-ROAD	✗	308	21	452	52	523	1035
KITTI-360	1190	711	999	1040	706	1048	1129
Total	8569	3085	2933	5529	7160	5149	10,486

Number of frames associated with each intersection class, for each of the used datasets. Please note that (i) in the KITTI-ROAD sequences, we did not add type-zero intersection frames, i.e., straight roads, to keep the balance between classes; furthermore, as our intentions are more focused on the understanding of real crossing context, this special kind of intersection has low relevance. (ii) The number of frames is not related at all with the number of different intersections, as multiple consecutive frames can be associated with the same area. Besides being recorded from a different position, we do not perform the same trajectory control procedures to avoid exact matching frames.

**Table 2:** Overall scheme of training approaches.

Learning Scheme	Key Features
Baseline	End-to-End Classification
	Standard RGB image classification method
Metric Learning	Learning to classify by using distances between
	embedding vectors
Teacher/Student	Two networks are sequentially trained
	metric learning can also be applied
LSTM	These networks are well suited to process time series data
PyTorchVideo	Deep Learning library for video understanding research

**Table 3:** Baseline results.

			ResNet					VGG			MobileNet-V3		Inception
			version					version			version		Version
			18	34	50	101	152	11	13	16	Large	Small	v3
KITTI-ROAD	RGB	Validation	0.69	0.71	0.69	0.72	0.70	0.58	**0.74**	0.73	0.66	0.69	0.65
		Test	-	-	-	-	-	-	**0.53**	-	-	-	-
	Warping	Validation	0.60	0.67	**0.70**	0.68	0.60	0.60	0.63	0.61	0.65	0.60	0.67
		Test	-	-	**0.62**	-	-	-	-	-	-	-	-
KITTI-360	RGB	Validation	0.83	0.82	0.77	0.81	0.81	0.80	0.84	0.81	0.82	0.73	**0.87**
		Test	-	-	-	-	-	-	-	-	-	-	**0.78**
	Warping	Validation	0.81	0.78	0.83	0.84	0.80	0.81	0.80	0.82	0.83	0.80	**0.87**
		Test	-	-	-	-	-	-	-	-	-	-	**0.70**
ALCALÁ	RGB	Validation	0.82	0.87	0.85	0.86	0.80	0.74	0.86	0.85	0.89	0.80	**0.89**
		Test	-	-	-	-	-	-	-	-	-	-	**0.94**
	Warping	Validation	0.88	0.91	0.82	0.90	0.88	0.85	0.89	0.85	**0.91**	0.87	0.91
		Test	-	-	-	-	-	-	-	-	**0.92**	-	-

Results using direct classification by the proposed architectures using RGB images and bird’s eye view images from the three selected datasets. Marked in bold are the best accuracy values for each type of data in both testing and validation.

**Table 4:** Teacher/student paradigm results.

			ResNet					VGG			MobileNet-V3		Inception
			Version					Version			Version		Version
			18	34	50	101	152	11	13	16	Large	Small	v3
KITTI-ROAD	RGB	Validation	0.51	0.50	0.53	0.50	0.57	0.66	0.70	0.71	**0.75**	0.71	0.57
		Test	-	-	-	-	-	-	-	-	**0.64**	-	-
	Warping	Validation	0.47	0.58	**0.70**	0.59	0.52	0.61	0.63	0.66	0.66	0.64	0.57
		Test	-	-	**0.61**	-	-	-	-	-	-	-	-
	3D-Images	Validation	0.45	0.55	0.65	**0.67**	0.58	0.56	0.62	0.60	0.60	0.62	0.46
		Test	-	-	-	**0.64**	-	-	-	-	-	-	-
	3D-Masked	Validation	0.64	0.71	0.72	0.71	0.71	0.70	0.72	0.72	**0.73**	0.72	0.69
		Test	-	-	-	-	-	-	-	-	**0.71**	-	-
KITTI-360	RGB	Validation	0.60	0.65	0.79	0.79	0.83	0.73	0.82	**0.86**	0.8	0.79	0.60
		Test	-	-	-	-	-	-	-	**0.75**	-	-	-
	Warping	Validation	0.63	0.61	0.69	0.8	0.63	**0.85**	0.79	0.83	0.82	0.73	0.70
		Test	-	-	-	-	-	**0.73**	-	-	-	-	-
	3D-Images	Validation	0.51	0.65	0.71	0.75	0.65	0.61	0.68	0.59	**0.79**	0.73	0.57
		Test	-	-	-	-	-	-	-	-	**0.73**	-	-
	3D-Masked	Validation	0.65	0.61	0.76	0.78	**0.79**	0.74	0.77	0.78	0.78	0.76	0.78
		Test	-	-	-	-	**0.67**	-	-	-	-	-	
ALCALÁ	RGB	Validation	0.76	0.49	0.88	0.88	0.86	0.82	**0.90**	0.87	0.88	0.77	0.81
		Test	-	-	-	-	-	-	**0.96**	-	-	-	-
	Warping	Validation	0.88	0.87	0.88	0.87	**0.91**	0.88	0.90	0.80	0.90	0.88	0.90
		Test	-	-	-	-	**0.90**	-	-	-	-	-	-

Results by dataset and type of data obtained using the Teacher/Student paradigm. Marked in bold are the best accuracy values for each type of data in both testing and validation.

**Table 5:** Metric learning paradigm results.

			ResNet					VGG			MobileNet-V3		Inception
			Version					Version			Version		Version
			18	34	50	101	152	11	13	16	Large	Small	v3
KITTI-ROAD	RGB	Validation	0.50	0.37	0.52	0.44	**0.54**	0.41	0.42	0.45	0.48	0.51	0.34
		Test	-	-	-	-	**0.56**	-	-	-	-	-	-
	Warping	Validation	**0.62**	0.47	0.40	0.35	0.46	0.37	0.41	0.35	0.47	0.56	0.38
		Test	**0.60**	-	-	-	-	-	-	-	-	-	-
	3D-Images	Validation	**0.49**	0.48	0.41	0.43	0.47	0.29	0.32	0.30	0.44	0.40	0.36
		Test	**0.47**	-	-	-	-	-	-	-	-	-	-
	3D-Masked	Validation	**0.65**	0.51	0.51	0.50	0.45	0.46	0.52	0.41	0.49	0.54	0.59
		Test	**0.72**	-	-	-	-	-	-	-	-	-	-
KITTI-360	RGB	Validation	0.32	0.64	0.64	0.59	**0.74**	0.59	0.23	0.25	0.55	0.53	0.72
		Test	-	-	-	-	**0.69**	-	-	-	-	-	-
	Warping	Validation	0.63	**0.65**	0.31	0.42	0.38	0.65	0.31	0.60	0.64	0.41	0.44
		Test	-	**0.78**	-	-	-	-	-	-	-	-	-
	3D-Images	Validation	0.57	0.53	0.29	0.40	0.62	0.36	0.42	0.33	**0.67**	0.56	0.60
		Test	-	-	-	-	-	-	-	-	**0.73**	-	-
	3D-Masked	Validation	0.65	0.61	0.76	0.78	**0.79**	0.74	0.77	0.78	0.78	0.76	0.78
		Test	-	-	-	-	**0.81**	-	-	-	-	-	
ALCALÁ	RGB	Validation	0.72	0.78	0.83	0.75	0.50	0.80	0.45	**0.84**	0.70	0.60	0.40
		Test	-	-	-	-	-	-	-	**0.94**	-	-	-
	Warping	Validation	0.74	0.81	0.72	0.52	0.81	0.71	0.77	0.59	**0.84**	0.83	0.82
		Test	-	-	-	-	-	-	-	-	**0.91**	-	-

Results by dataset and type of data obtained using the metric learning paradigm. For each type of data, we highlighted in bold the best MAPR for validation and the best accuracy for testing.

**Table 6:** Multi-frame paradigm results.

	KITTI ROAD		KITTI-360		Alcalá	
	Validation	Test	Validation	Test	Validation	Test
RGB	0.69	0.55	0.90	0.85	0.85	0.84
Warping	0.82	0.45	0.82	0.73	0.91	0.92
3D-Images	0.88	0.65	0.84	0.79	-	-
3D-Masked	0.90	0.70	0.89	0.81	-	-

Results by dataset and type of data obtained using temporal integration schemes. As the reader can see, the accuracies in testing greatly decrease in all configurations on KITTI and KITTI-360 datasets, but not with the Alcalá dataset. Finally, please notice that Alcalá dataset does not have 3D-Images and 3D-Masked values, as no stereo configuration and LiDAR data are available.

**Table 7:** Results with GAN-Augmented Dataset.

	Warped				RGB			
	Teacher/Student		Metric Learning		Teacher/Student		Metric Learning	
	Validation	Test	Validation	Test	Validation	Test	Validation	Test
ALL	0.890	0.894	0.901	0.872	**0.885**	0.894	**0.893**	0.817
ALL AUGMENTED (GAN)	**0.932**	**0.935**	**0.920**	**0.928**	0.876	**0.935**	0.882	**0.935**

Marked in bold are the best accuracy value for each type of data and each training methodology in both testing and validation.

## Data Availability

Regarding KITTI and KITTI360, the data presented in this study is openly available on http://www.cvlibs.net/datasets/kitti doi: 10.1177/0278364913491297 Access Date: 11 September 2021, and http://www.cvlibs.net/datasets/kitti-360 doi: 10.1109/CVPR.2016.401 Access Date: 11 September 2021; the Alcalá-1/Alcalá-2 datasets will be published on http://invett.es/intersectiondataset.
